# Stormwater Infrastructure Resilience Assessment against Seismic Hazard Using Bayesian Belief Network

**DOI:** 10.3390/ijerph20166593

**Published:** 2023-08-17

**Authors:** Maryam Garshasbi, Golam Kabir, Subhrajit Dutta

**Affiliations:** 1 Industrial Systems Engineering, University of Regina, Regina, SK S4S 0A2, Canada; maryam.garshasbi76@gmail.com; 2 Department of Civil Engineering, National Institute of Technology Silchar, Silchar 781017, India; subhrajit.nits@gmail.com

**Keywords:** resilience, stormwater pipe infrastructure, seismic hazard, Bayesian belief network, sensitivity analysis

## Abstract

Resilient stormwater infrastructure is one of the fundamental components of resilient and sustainable cities. For this, the resilience assessment of stormwater infrastructure against earthquake hazards is crucial for municipal authorities. The objective of this study is to develop a resilience assessment framework for stormwater pipe infrastructure against seismic hazards. A Bayesian belief network (BBN)-based stormwater infrastructure resilience model is constructed based on the published literature and expert knowledge. The developed framework is implemented in the city of Regina, Canada, to assess the city’s stormwater pipe infrastructure resilience. The outcome of the model indicates that proposed BBN-based stormwater infrastructure resilience model can effectively quantify uncertainties and handle the nonlinear relationships between several reliability and recovery factors. The model is also capable of identifying the most sensitive and vulnerable stormwater pipes within the network.

## 1. Introduction

Storm sewer drainage systems play a crucial role in preventing flooding by diverting excessive rain and groundwater that runs off impervious surfaces such as roofs, parking lots, sidewalks, and paved streets into nearby waterways through a network of drains and underground pipes [1]. These systems vary in design, ranging from simple residential drainage to complex municipal drains. It is important to manage the stormwater infrastructure system effectively as these infrastructures can be impacted severely due to natural hazards [2]. The potential natural hazards and their consequences on stormwater pipelines are mentioned in Table 1 [3].

During earthquakes, pipelines can be impacted in various ways, with the most common being shear forces resulting from fault movement and joint disconnection forces due to ground dislocation and liquefaction [4]. The ISO 16134 International Standard for Earthquake & Seismic Resilience recognizes ductile iron as the most resilient pipe material due to its high tensile strength, joint strength, and capacity to withstand deflection and strain. The development of new Earthquake Resistant Ductile Iron Pipe (ERDIP) systems that incorporate advanced connections with high axial ranges has further enhanced the resilience of pipelines to higher levels of seismic activity [3]. Pipeline failures during earthquakes are more widespread and frequent than commonly acknowledged and can seriously hinder emergency response efforts in some cases [5]. Earthquake damage to pipelines can result in an immediate breach or cause stress to accumulate at specific points along the line, leading to a breach days or even weeks after the earthquake, despite the surface appearing stable [6]. Therefore, it is vital to develop a resilient stormwater infrastructure system.

Bruneau et al. [7] and Cimellaro et al. [8] defined physical and social systems’ resiliency with four “R”, which are robustness, redundancy, resourcefulness, and rapidity. The ability of infrastructure to withstand stress and continue functioning without losing critical functions is known as robustness, which is a crucial component of resilience. This means that a network’s ability to function even after being exposed to external pressures or disruptions is an important aspect of its robustness. Redundancy allows for alternative options, decisions, and substitutions within a system to provide different recovery options in the event of a disaster. Resourcefulness refers to the capacity to manage the aftermath of a disaster on the system, such as mobilizing essential workers, operations, and materials for rapid recovery. Rapidity measures how quickly the network can be repaired and restored to its original function after a hazard, and is a critical component of resilience [9].

**Figure 1:**
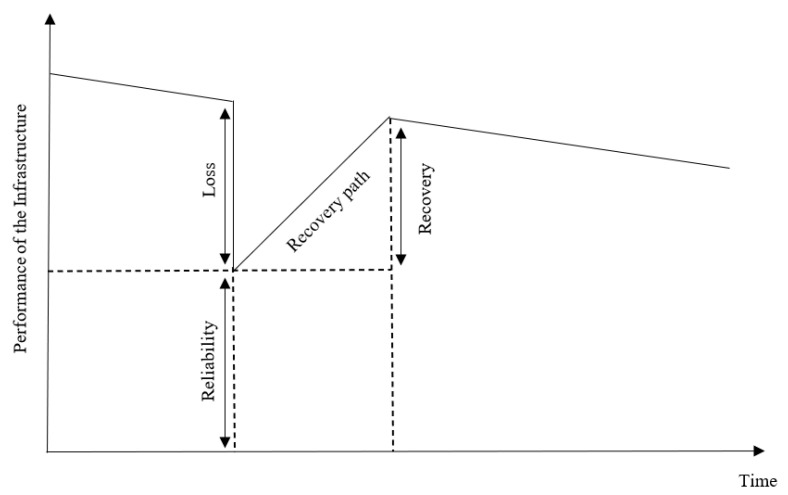
Infrastructure’s performance throughout the service life (modified after Sen et al. [10]).

In this context, it is important to note that robustness is a subset of reliability, whereas resourcefulness, rapidity, and redundancy are subsets of recovery. As a result, this study considers reliability and recovery as the primary factors for evaluating resilience [10]. The life cycle of an infrastructure can be represented in Figure 1, which displays its performance before, during, and after a hazard. This diagram provides an overview of the infrastructure’s service life and how it performs in the event of a significant failure or disaster.

Figure 1 presents the infrastructure system’s performance. The performance of an infrastructure system gradually deteriorates over time due to its usage. In the event of a disaster, there is a sudden decline in performance, referred to as the failure path or loss, which is determined by the type of catastrophe and the system’s robustness. The recovery time and path, on the other hand, are dependent on the type of infrastructure system and the availability of resources. It is uncertain how the failure and recovery path will unfold [10]. 

Since the concept of resilience has been introduced in stormwater systems, resilience measures have been recognized as crucial in the decision-making process for developing strategies to prepare for, respond to, and recover from unexpected disruptive events affecting water infrastructure systems. Conducting a stormwater resilience analysis can offer several benefits, including identifying and comparing the resilience of systems under different organizational, economic, environmental, and social conditions; identifying vulnerable areas that require improvement through resilience strategies; and enhancing clarity in the design of infrastructure systems [11,12].

Therefore, the main research aim of this study is to develop a stormwater pipe resilience model against earthquakes to improve the management of the stormwater pipelines. The specific highlighted objectives and contributions of this study are as follows:

1. To create a stormwater pipeline resilience assessment model considering safety and recovery criteria.
2. To apply the Bayesian belief network (BBN) method for robust modeling of the causal relationship between the resilience indicators.
3. To evaluate recovery and reliability factors of stormwater pipe infrastructure system and evaluate the sensitivities for informed decision making on pipeline management.

The remainder of this study is organized into four sections. The literature is reviewed in Section 2. Section 3 shows the research methods that have been used in this study. Data collection is explained in Section 4. In the last chapter, which is Section 5, the development of the BBN model in this study is shown.

## 2. Literature Review

The first section of this piece of writing discusses the various methods used to determine infrastructure resiliency, while the second section presents a literature review related to the resilience of water pipe infrastructure.

### 2.1. Infrastructure Resilience Methodology 

In various studies, different methodologies have been employed to investigate infrastructure resilience. For instance, Murdock et al. [13] utilized a response curve approach to assess the resilience of critical infrastructure to flooding. Meanwhile, Muller [14] proposed a fuzzy-rule-based approach to select alternative architectures in an interconnected infrastructure system to enhance overall system resilience. This method takes into account the most important factors in decision making for resilience strategies. The study concluded by proposing a method based on existing resilience architecting strategies that combine essential system aspects using fuzzy memberships and fuzzy rule sets.

Rehak et al. [15] proposed a method called the Critical Infrastructure Elements Resilience Assessment (CIERA) for assessing the resilience of critical infrastructure elements. The approach involves a statistical evaluation of the elements’ robustness, ability to recover from disruptive events, and capacity to adapt to past experiences. This method includes assessing both technical and organizational resilience and identifying weaknesses that need to be addressed to improve resilience. In another study, Yuan et al. [16] emphasized the importance of critical infrastructures, such as road networks, in providing transportation to hospitals and shelters during disasters. They proposed an Internet of People-enabled framework to evaluate the performance failure of road networks during disasters and provide a performance failure rate as a measure of road network resilience.

### 2.2. Water Pipe Infrastructure Resilience

The importance of resilience in water infrastructure systems is emphasized by identifying its critical features, such as being prepared for hazardous situations and considering its interdependence with the electrical infrastructure. This is done to enable water infrastructure system managers to improve the system’s resilience. Matthews [17] conducted research on this topic, which included describing and quantifying critical characteristics of water infrastructure system resilience, such as redundancy in water systems and storage in wastewater systems. Other studies, such as those conducted by Cimellaro et al. [18] and Ouyang and Dueñas-Osorio [19], also emphasize the importance of understanding resilience in water infrastructure systems. The operation of water distribution systems can be disrupted by various types of hazards and disasters, whether they are natural or caused by human activity. Natural hazards are physical events that can occur quickly or slowly over time. These hazards are categorized into different types as shown in Table 2 [20]. In the following subsections, the literature related to hazards for water infrastructure is shown in detail.

### 2.3. Natural Hazards

Quitana et al. [21] conducted research on the ability of drinking water systems to withstand natural hazards, focusing on the resilience of critical infrastructure. Stip et al. [22] carried out a study aimed at informing water system managers about the importance of, and strategies for, increasing the resilience of water service infrastructure to natural hazards and climate risks. Stip et al. [22] also mentioned that, in selecting resilience measures, water systems managers have to take into account six principles and incorporate the decision-making concept under deep uncertainty.

#### 2.3.1. Earthquake

Developed countries possess better protection against catastrophic disasters than developing countries due to their abundance of financial and technological resources, as well as their organized design codes and administration processes. Infrastructure systems in developing countries are more vulnerable to catastrophic disasters [4,23]. Nazarnia et al. [23] evaluated the infrastructure resilience in developing countries by focusing on the water system in the Kathmandu Valley after the 2015 Nepalese earthquake. They created a framework for the systemic evaluation of infrastructure resilience to assess the water supply system in that area. Similarly, Mostafavi et al. [24] studied the resilience of the water infrastructure in the Kathmandu Valley following the 2015 Nepalese earthquake using a system approach. Mostafavi et al. [24] identified the factors and their relationships that impacted the resilience of the Kathmandu Valley water system. The research findings underscored the factors that decreased resilience in the system, including the supply–demand imbalance, aging infrastructure, and a lack of disaster management procedures.

#### 2.3.2. Flood and Coastal

The water infrastructure in coastal areas is highly susceptible to climate-sensitive hazards such as salt intrusion, rainfall, tides, and storm surges, which can have detrimental impacts on both infrastructure and human health. In their study, Allen et al. [25] investigated the increasing frequency, magnitude, and consequences of flooding hazards on water infrastructure and public health due to rising sea levels.

#### 2.3.3. Weather–Climate

Falco and Webb [26] explained that extreme weather events, including rising global temperatures and climate change, can have significant impacts on water infrastructure, leading to consequences such as disrupted clean water distribution, wastewater treatment, and stormwater control. Hossain et al. [27] defined weather–climate-resilient water infrastructure as infrastructure capable of forecasting, adapting to, and recovering from external disruptions caused by adverse weather and climate conditions and providing necessary services. Stip et al. [22] conducted a study to advise water system managers on strategies for enhancing the resilience of water service arrangements to natural hazards and climate risks.

### 2.4. Terrorist Attacks

In a society disturbed over the possibility of terrorism, the privacy and security of infrastructure data are critical. Nevertheless, the study on infrastructure security is complex in this situation because searches on real systems cannot be announced. “Virtual cities” are one potential key to this issue, and a library of these virtual cities is now under extension [28]. Brumbelow et al. [28] conducted a study about virtual cities for water distribution and infrastructure systems. 

The reviewed studies are presented in Table 3, covering various water infrastructure systems. However, there is a lack of literature specifically addressing the resilience of stormwater pipe infrastructure to earthquake hazards. This thesis aims to fill this gap by focusing on the resilience of stormwater pipes and their contributing factors against earthquake hazards, making it a unique contribution to the existing literature.

### 2.5. Research Gap

In most of the earlier analyses, the earthquake resilience of stormwater pipelines is not highlighted comprehensively, considering the safety and recovery criteria. Studying earthquake resilience of stormwater pipelines is important because with that study, most influential factors, their weights, and their importance can be determined, and executors/utility engineers can use the information to make the infrastructure more resilient against earthquake hazards. The majority of the previous studies focused primarily on water distribution systems, with very few on stormwater pipeline failure and resilience. Studying the resiliency of the stormwater system is as important as other water systems because if a stormwater pipe cracks and fails after an earthquake hazard, the extra runoff that does not soak into the ground will not go to the stormwater pipe system and will cause flooding. There were few analyses regarding the resiliency, reliability, and recovery factors of stormwater pipes, as most of the previous studies mainly focused on risks and not resiliency. In this study, the Bayesian belief network (BBN) method has been used for probabilistic modeling and the quantification of resilience for stormwater pipeline systems engineering with limited information. Furthermore, a geographic information system (GIS) tool is implemented in this research for data inventories, visualization, and evaluations.

## 3. Methodology 

**Figure 2:**
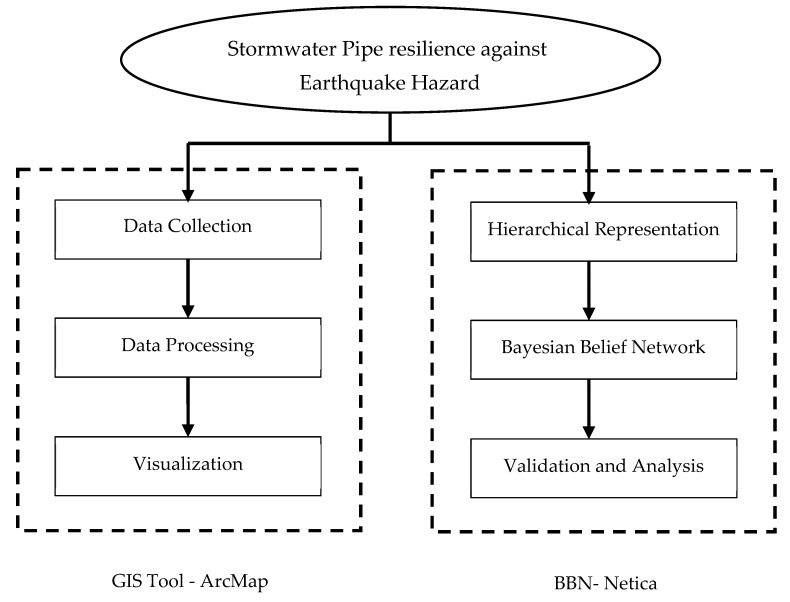
Workflow process.

A novel approach is proposed in this study to address the limitations of existing seismic resilience methods. The proposed method is a combination of ArcGIS and BBN and can capture the interrelationships between different factors, measure uncertainty, and integrate data and knowledge-based sources. Figure 2 demonstrates the process workflow of this approach.

### 3.1. Infrastructure Resilience Parameter Identification

**Figure 3:**
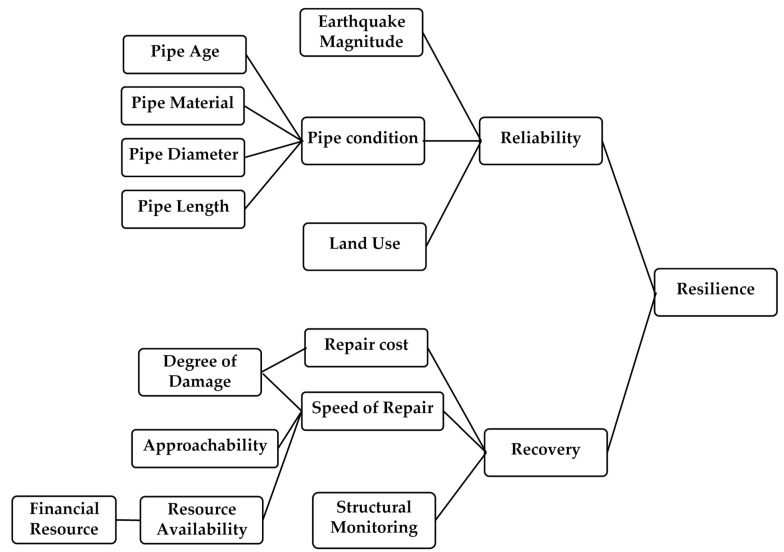
Hierarchical representation of stormwater pipe’s resilience against earthquake hazards.

Physical and social systems resiliency can be defined by robustness, redundancy, resourcefulness, and rapidity [7,8,29]. In the first step, essential factors and the following parameters must be selected in order to develop the framework for stormwater infrastructure resiliency against earthquakes. Figure 3 highlights the critical parameters for stormwater pipeline resilience against earthquake hazards.

### 3.2. Hierarchical Model Development 

In this study, resilience is connected to the reliability and recovery of the infrastructure [10,29]. Figure 3 highlights the hierarchical representation of the resilience framework. Based on Figure 3, the reliability of this infrastructure is connected to three main factors: pipe condition, earthquake magnitude, and the land use type the pipe is located in. The four factors that determine the condition of a pipe are its age, material, diameter, and length. Research by Dong and Frangopol [30] and Vishwanath and Banerjee [31] suggests that newly installed pipes are better able to withstand earthquakes than older pipes, and that stronger materials provide more reliability against earthquakes. Regarding earthquake magnitude, it is obvious: higher magnitudes will cause more damage to the pipe. 

Additionally, there are three key elements associated with the process of recovery, which include the cost of repairs, the rate at which repairs are completed, and the monitoring of the structure. Regular structural monitoring of stormwater pipes can help in swiftly diagnosing issues and determining the condition of the pipes after incidents, ultimately leading to a faster recovery process. This has been noted in studies conducted by Gay and Sinha [32] and Mebarki et al. [33]. 

Repair cost depends on the degree of damage [34]. The rate at which repairs can be completed is determined by three factors: the extent of the damage, the availability of resources, and the accessibility to those resources. In particular, the recovery process may be delayed if there is a lack of pipe materials during or after a disaster, as noted in the study by Sen et al. [10]. Similarly, disturbances that affect the accessibility of resources can also slow down the recovery process.

### 3.3. Bayesian Belief Network (BBN)

A BBN is a set of graphical models that gives a slight description of the probabilistic dependencies among a provided collection of random variables [35]. A BBN can also be described as a graph-based model containing edges and nodes. Nodes in a BBN outline model variables and edges that describe the relationships among the nodes and also the conditional dependencies [35,36]. In a BBN, the nodes are classified into a finite collection of variables with their probability amounts [37]. 

A BBN is a directed acyclic graph which includes a set of vertices which are variables or nodes and a set of edges which are arcs or links. As mentioned earlier, a BBN is a graphical model for reasoning under uncertainty where the nodes $V=\left(X_{1},\;X_{2},\;X_{3},\ldots ,\;X_{n}\right)$ describe random variables and the links describe conditional dependencies among the variables [38,39]. The strength of the dependencies between variables and their parent nodes in a Bayesian network is determined by the conditional probability tables (CPTs) assigned to each dependent node (or child). These tables outline the probability of a child node being in a certain state given any combination of parent node states. This allows for the calculation of the likelihood of certain events or outcomes based on the probabilities of related variables in a given scenario [39].

The conditional probability distribution (CPD) of the variable *Xi* delivered by its direct parents’ node is expressed as *P*(*Xi|pa*(*Xi*)), of which *pa(Xi)* is a set of all parents of the variable $X_{i}$. The joint probability distribution of each variable defined in *V* and *P(V)* will be created from the CPDs as:

(1) $$P\left(V\right)=P\left(X_{1},\;X_{2},\ldots ,\;X_{n}\right)\;=P\left(X_{1}|pa\left(X_{1}\right)\right)\times \;P\left(X_{2}|pa\left(X_{2}\right)\right)\times \ldots \times P(X_{n}|pa(X_{n}))\;=\prod_{i=1}^{n}P(X_{i}|pa(X_{i})).$$

Probability updating is one of the critical features that a BBN controls. It allows the decision makers to refresh the probability of the variable *P*(*Xi*), providing a new view named evidence *e*. The updated probability can be presented as

(2) $$P\left(V|e\right)=\frac{P\left(V,\;e\right)}{P\left(e\right)}=\frac{P\left(V,e\right)}{\sum_{V/e}P\left(V,e\right)}$$

where *P*(*V|e*) is the updated joint probability; $\underset{V/e}{\sum }\left(.\right)$ is the summation over all values of *V* except *e* [40].

### 3.4. Data Collection

The goal of this study is to find influential factors that can guarantee storm pipeline infrastructures’ resilience against earthquake hazards and, after that, find out the recovery, reliability, and resiliency index for each of the pipes to see how much the infrastructure will be resilient in the aftermath of seismic hazards. To this end, the first thing that needs to be done is the finding of the influential factors on recovery, reliability, and resiliency or, in other words, the input data to the proposed model along with their scales and states. This study utilized expert judgment and input from experienced professionals to identify the most significant inputs. The experts were chosen from a pool of academics and engineers with expertise in the area of stormwater pipeline assessment, and their information is presented in Table 4. Initially, the academics provided guidance on relevant inputs and potential outcomes based on the existing literature in the field. From there, the input of the experienced professionals was gathered to further refine the most important variables.

#### 3.4.1. Input Factors

Figure 3 illustrates a hierarchical representation of the resilience factors associated with stormwater pipes in the context of earthquake hazards, along with the specific factors involved. Table 5 presents the finalized input factors for assessing the resilience of stormwater pipes against earthquakes, including their respective states and scales. The inputs used in this study are pipe age, pipe material, pipe diameter, pipe length, land use type, earthquake magnitude, financial resources, approachability, structural monitoring, and degree of damage.

**Figure 4:**
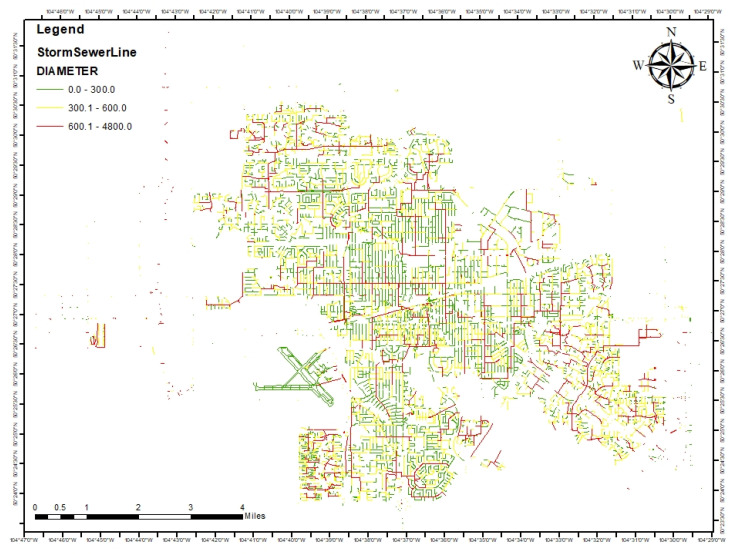
Pipe diameter differences in the city of Regina.

The age, material, and diameter of stormwater pipes are important factors that affect their resiliency. Pipe age is categorized as new (excellent), moderate, or old (poor). Different pipe materials, such as PVC, CSP, AC, Steel, RCP, CONC, and TILE, are also categorized as poor, moderate, or excellent. Pipe diameter is used to determine the flow through the pipes and is categorized as large (excellent), medium (moderate), or small (poor). Figure 4 shows the variation of pipe diameter in the city of Regina with different colors, where green pipes indicate smaller diameter and red pipes indicate larger diameter.

The pipe length is assessed by m, and small (excellent), medium (moderate), and large (poor) scales are considered for them. Land use refers to the purpose for which an area is being used and its impact on the consequences of pipe failure. The consequences of a pipe failure on land used for commercial or institutional purposes will be more severe than on open spaces or railways. The quality of land use is classified as poor, moderate, or excellent.

The earthquake magnitude is another factor that affects the model’s input. Table 5 was created based on Michigan Tech [42] and Track [43] to list the different states and scales of earthquake magnitudes. Although approachability and financial resources are in good condition in the city of Regina, they are important factors to consider in recovery processes and will be evaluated in different scenarios and states. Structural monitoring, unfortunately, is not in good condition in the city of Regina. The level of damage is another recovery factor that will be evaluated in various scenarios.

#### 3.4.2. Dependent Factors

In this study, seven dependent factors are finalized by assessing the different consequences due to the failure of pipes. The dependent factors and their states are provided in Table 6.

### 3.5. BBN Model Development

**Figure 5:**
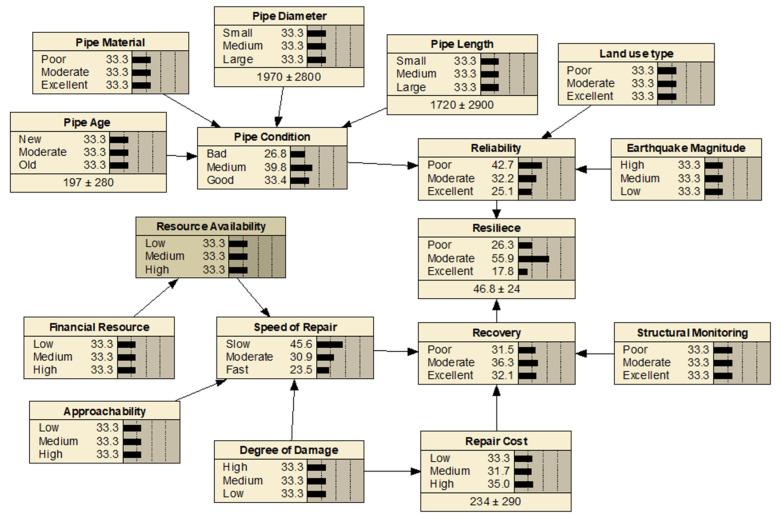
Proposed BBN model for the resiliency of stormwater pipe.

For the BBN model development, five experts participated in the process. Initially, the suitable and appropriate criteria and sub-criteria for the stormwater pipe resilience framework have been presented in Table 5 and Table 6. Then, the parent and child nodes have been verified to develop the causal diagram. Figure 5 illustrates the proposed BBN model for the resiliency of stormwater pipes where 10 parent or independent nodes and 7 child or dependent nodes are connected with 17 links. The BBN model structure development was accomplished using Netica software Version 5.0 [44].

The dependencies between the child nodes and the parent nodes were quantified using CPT. The CPT values of the child nodes were based on expert judgment or knowledge elicitation. Table 7 presents an example (node: *speed of repair*) of determining the conditional probabilities based on knowledge elicitation. The *speed of repair* child node depends on the *resource availability, approachability*, and *degree of damage* parent nodes. According to Table 7, if the *resource availability* and *approachability* are low and the degree of damage is low, the corresponding CPT values for *speed of repair* are 98, 2, and 0, which indicate that the conditional probabilities for *speed of repair* being in the state of slow, moderate, and fast are 98.0%, 2.0%, and 0%, respectively. The CPTs of the other child nodes were calculated in a similar way. The Netica considers uniform probabilities for missing entries or for nodes whose CPTs are incomplete or absent.

### 3.6. Model Validation

For validating the proposed model, a set of qualitative and quantitative validation procedures have been done in the following subsections. 

#### 3.6.1. Extreme Condition Test

The study analyzed two extreme conditions to assess the resilience of stormwater pipe infrastructure. Extreme A represents the best possible states of all the parent nodes, while Extreme B represents the worst possible states of all the parent nodes. The developed BBN framework was applied to both scenarios, and the results are presented in Table 8.

#### 3.6.2. Scenario Analysis

In a scenario analysis, various hypothetical scenarios are taken into consideration instead of two extreme conditions. Table 9 shows the states of the parent nodes and the resilience probability distribution for five scenarios. 

In Scenario 1, all the parent nodes are in the best states except pipe age, which is in a poor condition (old). In this scenario, the resiliency decreased from 82.2 ± 11 to 75.2 ± 17. Scenario 2 is same as scenario 1, except the pipe age is in a moderate condition. It is shown that the resiliency increased from 75.2 ± 17 to 81.1 ± 13 in comparison with scenario 1. In scenario 3, all the parent nodes are in the best condition except high earthquake magnitude and high degree of damage. In this case, the resiliency decreases from 82.2 ± 11 to 58.1 ± 24. 

Scenario 4 has a different status to scenario 1 to 3. In scenario 4, all the parent nodes are considered in the worst condition except high financial resources and approachability. It shows an increase in resiliency from 19.8 ± 14 to 30.5 ± 19. In scenario 5, same as scenario 4, all the parent nodes are considered in the worst condition but in this scenario the structural monitoring considered as excellent. The resiliency number in scenario 5 increased from 19.8 ± 14 to 22.1 ± 16. 

#### 3.6.3. Sensitivity Analysis

**Figure 6:**
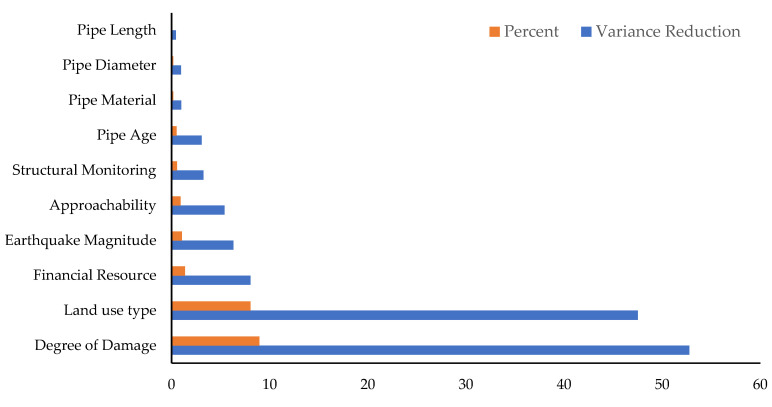
Sensitivity analysis of the proposed resilience framework for stormwater pipe infrastructure.

This study includes sensitivity analysis to identify critical factors for stormwater pipe infrastructure resilience and to quantitatively validate the model. Sensitivity analysis provides information on how sensitive the model’s outputs are to small changes in uncertain input variables. The variance reduction technique is used in this study to determine the sensitivity of the BBN model. This technique calculates the variance reduction of the expected value of a query node (resilience) resulting from a change in a variable node (such as age, diameter, or length). This approach has been used in previous studies [45,46,47] and is employed here to validate the proposed model. Therefore, the variance of the actual value of R given the evidence O and V(R/o) is calculated employing the subsequent equation [44,48]:

(3) $$V\left(R|o\right)=\sum_{𝓏}p\left(r|o\right)\left[Y_{r}-E\left(R|o\right)\right]{|}^{2}$$

where *o* is the state of the varying variable node *O*, *r* is the state of the query node *R*, *p*(*r/o*) is the conditional probability of *r* given *o*, $Y_{r}$ is the numeric value corresponding to state *r*, and *E*(*R*/*o*) is the expected real value of *R* after the new finding *o* for node *O* [46]. The sensitivity analysis outcomes by the parent nodes for the child node resiliency are shown in Table 10 and summarized in Figure 6.

According to the Table 10, *degree of damage* showed the highest contribution to variance (8.94%), followed by *land use type* (8.05%). Being the most sensitive parameters, changes in *degree of damage* and *land use type* have the substantial effect on the final output *resiliency*. To a lesser degree, *financial resources*, *earthquake magnitude*, and *approachability* show sensitivities of 1.36%, 1.06%, and 0.915%, respectively. Finally, *structural monitoring*, *pipe age*, *pipe material*, *pipe diameter*, and *pipe length* showed lower sensitivity in the range of 0.07% to 0.55%. 

## 4. Results and Discussions

**Figure 7:**
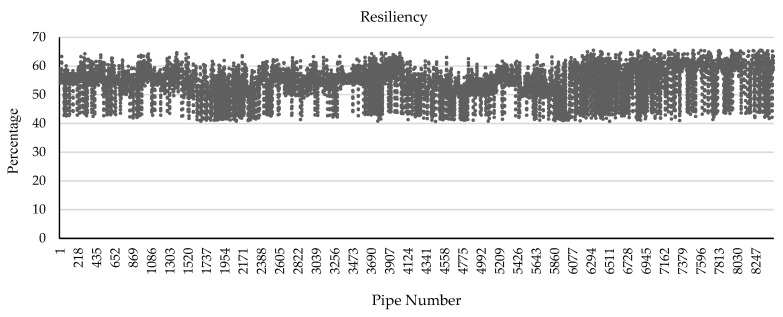
The sparsity of resiliency of stormwater pipes of city of Regina.

Table 11 indicates five samples of stormwater pipe characteristics and corresponding resiliency. Similarly, the resiliency of the 8464 stormwater pipes of the city of Regna is defined using the developed BBN model. Figure 7 shows the sparsity of resiliency of stormwater pipes in the city of Regina. Pipe age, material, diameter, length, and land use type are unique for each and every pipe in this model. On the other hand, constant states were considered for other factors, which were the same in the whole city. Earthquake magnitude is considered medium in the city, financial resources and approachability are considered high, degree of damage is considered medium, and structural monitoring is considered poor. As it is obvious from Figure 7 and the data, the resiliency numbers are between 40.60 and 65.56 percent.

**Figure 8:**
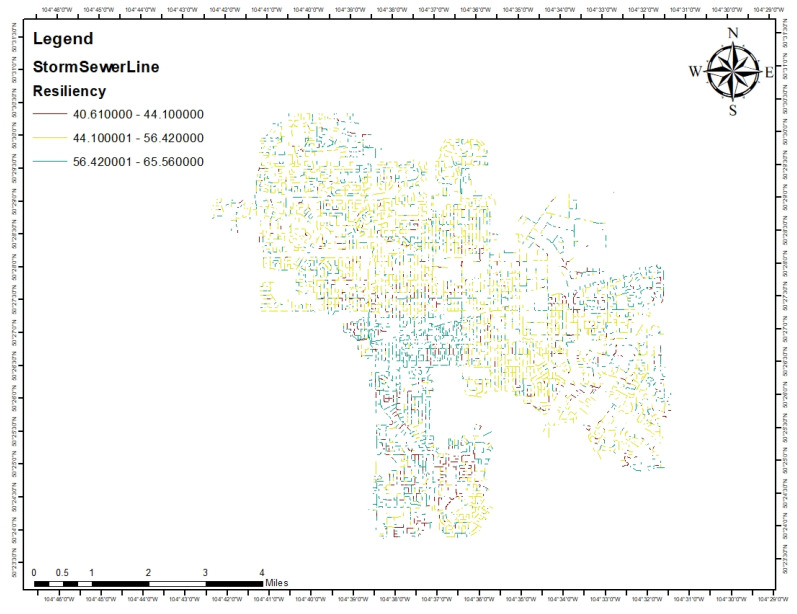
The resiliency of the city of Regina’s stormwater pipes.

Figure 8 presents the resiliency of the city of Regina’s stormwater pipes. The more the color of the feature tends to be green, the more resilient it is. On the basis of Table 6, a resiliency between 34% to 66% is considered moderate resiliency. Based on the inputs that were put, in the whole city, for medium earthquake magnitude, the resiliency is moderate for all of the stormwater pipes in the city of Regina.

## 5. Conclusions

This study gives a framework for assessing the city of Regina’s stormwater pipe resilience against earthquake hazards by using a BBN approach. To this end, the actual data from a stormwater pipe system of Regina in Canada have been obtained from the open data website of the city of Regina. Data have been opened and extracted through ArcGIS (ArcMap 10.5.1) software. To assess stormwater pipes’ resiliency against the earthquake hazard, finding the factors and the relationships between them is the initial priority. After determining the stormwater pipes’ resilience, three distinct performance states for each of the pipes were generated. The belief values computed through the proposed framework can indicate the potential resilience of a stormwater pipe towards earthquake hazards.

As one of the conclusions of this study, between reliability and recovery, resiliency is more sensitive to reliability than recovery. With regard to independent factors, the degree of damage factor is considered as the most sensitive factor in comparison with other factors in finding the resiliency of the stormwater pipe system of the city of Regina. Based on Figure 8, and all the inputs that were put in the model in the whole city for a medium earthquake magnitude, the resiliency is moderate for all of the stormwater pipes in the city of Regina as the resiliency for all of the pipes is between 40.60 and 65.56 percent. 

The analysis output will aid in identifying the crucial factors by assessing the resiliency of each pipe in the entire system, allowing the agency to promptly address the critical factors and improve their strength, thereby enhancing the resilience of the stormwater pipe infrastructure. Thus, the study’s results can accurately help decision makers determine the resiliency of the stormwater pipe infrastructure. The study’s findings and figures can guide the management in preparing for potential earthquake hazards and recovering from such events. By identifying the most resilient pipes, the managers can improve the resilience of other pipes to withstand future earthquake hazards.

The effectiveness of the model is limited by the accuracy of the information and opinions provided by the experts regarding the related factors and their relationships. Thus, it is recommended to establish a global network system with the collaboration of experts from earthquake-prone countries, such as Japan and Turkey, to ensure the comprehensive and accurate evaluation of the model.

For further research, the current framework for resilient stormwater pipe infrastructure can be expanded to encompass other hazards, such as droughts, floods, landslides, climate change, and tsunamis. Furthermore, a more comprehensive framework that considers vulnerability, robustness, and recovery, with more complex dependencies at the factor level, can be developed. Other mathematical theories of uncertainty, such as rough sets theory and fuzzy sets theory, could also be utilized to assess resiliency. To validate the BBN method, the simple multi-attribute rating technique (SMART) or Dubois and Prade’s method for the compound rule can be used for examining multiple criteria. Furthermore, a similar assessment can be applied to provide a consequence model for various buried infrastructures, such as oil and gas pipelines and drinking water, in future studies. 

## Figures and Tables

**Table 1:** Potential natural hazards and their consequences on pipelines (modified after Mundy [3]).

Natural Hazards	Consequences
Earthquakes	Breaks in pipes, outage of power
Floods	Disruption in service including stormwater, treatment, source water, distribution, or storage
Tornadoes	Loss of pressure/leaks
Drought	Other infrastructure damage or failure
Tsunamis	Change in the quality of water
Hurricanes	Failure in access to facilities and supplies
Wildfires	Environmental consequences
Winter Storms	Financial influence, such as loss of revenue or repair costs

**Table 2:** Natural hazards [20].

Natural Hazards	Examples
Geophysical	Volcanic activity, earthquakes, landslides, and tsunamis
Climatological	Wildfires, extreme temperatures, and drought
Biological	Insect/animal plagues and disease epidemics
Hydrological	Floods and avalanches
Meteorological	Cyclones and wave/storm surges

**Table 3:** List of studies related to natural hazards.

	Hazards for Water Infrastructures				
Reference	Natural Hazards	Weather–Climate	Earthquake	Flood and Coastal	Terrorist Attacks
Brumbelow et al. [28]					√
Hossain et al. [27]		√			
Falco and Webb [26]		√			
Nazarnia et al. [23]			√		
Mostafavi et al. [24]			√		
Stip et al. [22]	√	√			
Allen et al. [25]				√	
Quitana et al. [21]	√				

**Table 4:** Details of the Experts.

Expert No.	Profile	Experience	Roll in Work/Designation
Expert 1	Ph.D., P.Eng.	12 years	Assistant Professor
Expert 2	Ph.D.	18 years	Associate Professor
Expert 3	B.Sc., P.Eng.	33 years	Manager, Water and Sewer Engineering at City of Regina
Expert 4	Senior Engineer	25 years	Senior Engineer at City of Regina
Expert 5	MASc, P.Eng.	8 years	Engineer, Ministry of Highway, SK

**Table 5:** Inputs, scales, and the state of the model (modified after Garshasbi and Kabir [41]).

No.	Inputs	Scales	States
Reliability Factors			
1	Pipe age (years)	Age > 60	(Old) Poor
		30 < age ≤ 60	Moderate
		Age ≤ 30	(New) Excellent
2	Pipe material	Poly—Poly B ^1^, Preload (a temporary thing)	Poor
		AC ^2^, CONC ^3^, CL ^4^, Tile, TR ^5^, VCT ^6^	Moderate
		RCP ^7^, PVC ^8^, PVC ribbed, PVC permalock, corrugated galvanized steel, HDPE ^9^, CSP ^10^, perforated poly, steel	Excellent
3	Pipe diameter (mm)	Diameter ≤ 300	(Small) Poor
		300 < Diameter ≤ 600	(Medium) Moderate
		Diameter > 600	(Large) Excellent
4	Pipe length (m)	Length > 100	(Large) Poor
		50 < Length ≤ 100	(Medium) Moderate
		0 < Length ≤ 50	(Small) Excellent
5	Land use	Commercial, institutional	(High) Poor
		Residential (HD ^11^, MD ^12^, LD ^13^), industrial (HI ^14^, LI ^15^, MI ^16^),	(Medium) Moderate
		Railway, contract zone, contract, open space/recreation, urban holdings	(Low) Excellent
6	Earthquake magnitude	Magnitude > 7	(High) Poor
		6 < Magnitude ≤ 7	(Medium) Moderate
		Magnitude ≤ 6	(Low) Excellent
Recovery Factors			
7	Approachability	No route available	(Low) Poor
		Alternate route available	(Medium) Moderate
		No problem in approachability	(High) Excellent
8	Financial resources	No	Low (Poor)
		Yes, but not received	(Medium) Moderate
		Yes, and received	(High) Excellent
9	Structural monitoring	No	Poor
		Yes, every 5 years	Moderate
		Yes, every year	Excellent
10	Degree of damage	Broken and needs pipe replacement	(High) Poor
		Moderately damaged pipe and repairable	(Medium) Moderate
		No damage	(Low) Excellent

1: Poly—Poly B: polybutylene; 2: AC: asbestos cement; 3: CONC: concentric; 4: CL: chlorine; 5: TR: improved thermoplastic rubber; 6: VCT: vinyl composition tile; 7: RCP: rigid concrete pipe; 8: PVC: polyvinyl chloride; 9: HDPE: high-density polyethylene; 10: CSP: corrugated steel pipe; 11: HD: high density; 12: MD: medium density; 13: LD: low density; 14: HI: heavy industrial; 15: LI: light industrial; 16: MI: medium industrial.

**Table 6:** Dependent factors and their states and scales (modified after Sen et al. [10]).

No.	Factor	Scales	States
1	Pipe condition	-	Poor
			Moderate
			Excellent
2	Repair cost (thousand $)	Cost >160	(High) Poor
		16 < Cost ≤ 160	(Medium) Moderate
		0 < Cost ≤ 16.	(Low) Excellent
3	Speed of repair	-	(Slow) Poor
			Moderate
			(Fast) Excellent
4	Resource availability	Not available.	(Low) Poor
		Yes, but cost of the resources increased by <10%.	(Medium) Moderate
		Yes, but cost of the resourceincreased by >10%.	(High) Excellent
5	Reliability	The infrastructure is fully damaged.	Poor
		Damage occurs to the pipeline and cracks and leaks will happen.	Moderate
		Minor damage happens to the infrastructure and causes minor leaks.	Excellent
6	Recovery	Takes more than 35 days for recovery.	Poor
		Takes 11 to 35 days for recovery.	Moderate
		Takes 10 or less than that for recovery.	Excellent
7	Resilience	0 to 33% probability that the pipe can withstand or go back to its original level.	Poor
		34 to 66% probability that the pipe can withstand or go back to its original level.	Moderate
		67 to 100% probability that the pipe can withstand or go back to its original level.	Excellent

**Table 7:** Sample conditional probability tables for the child node *Speed of Repair*.

Resource Availability	Approachability	Degree of Damage	Speed of Repair		
			Slow	Moderate	High
Low	Low	High	98	2	0
Low	Low	Medium	90	10	0
Low	Low	Low	80	20	0
----	----	----	----	----	----
----	----	----	----	----	----
Medium	Medium	High	45	55	0
Medium	Medium	Medium	15	70	15
Medium	Medium	Low	10	55	35
----	----	----	----	----	----
----	----	----	----	----	----
High	Medium	Low	0	15	85
High	High	High	0	30	70
High	High	Medium	0	15	85
High	High	Low	0	2	98

**Table 8:** Extreme condition test for the proposed BBN model.

Node	Extreme A	Extreme B
Pipe age	New	Old
Pipe material	Excellent	Poor
Pipe diameter	Large	Small
Pipe length	Small	Large
Land use type	Excellent	Poor
Earthquake magnitude	Low	High
Financial resources	High	Low
Approachability	High	Low
Degree of damage	Low	High
Structural monitoring	Excellent	Poor
Reliability	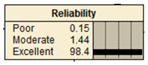	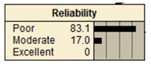
Recovery	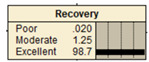	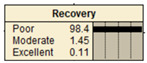
Resiliency	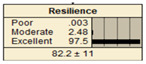	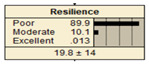

**Table 9:** Scenario analysis for the proposed BBN model.

Node	Scenario 1	Scenario 2	Scenario 3	Scenario 4	Scenario 5
Pipe age	Old	Moderate	New	Old	Old
Pipe material	Excellent	Excellent	Excellent	Poor	Poor
Pipe diameter	Large	Large	Large	Small	Small
Pipe length	Small	Small	Small	Large	Large
Land use type	Excellent	Excellent	Excellent	Poor	Poor
Earthquake magnitude	Low	Low	High	High	High
Financial resources	High	High	High	High	Low
Approachability	High	High	High	High	Low
Degree of damage	Low	Low	High	High	High
Structural monitoring	Excellent	Excellent	Excellent	Poor	Excellent
Resiliency	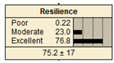	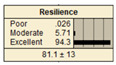	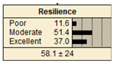	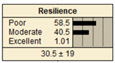	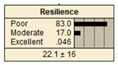

**Table 10:** Sensitivity analysis of *resiliency* node.

Node	Variance Reduction	Percent	Mutual Info	Percent	Variance of Beliefs
Degree of damage	52.77	8.94	0.0802	5.55	0.0062
Land use type	47.52	8.05	0.0742	5.14	0.0048
Financial resources	8.032	1.36	0.0117	0.811	0.0008
Earthquake magnitude	6.288	1.06	0.0092	0.635	0.0006
Approachability	5.405	0.915	0.0079	0.545	0.0006
Structural monitoring	3.245	0.55	0.0047	0.326	0.0003
Pipe age	3.056	0.517	0.0045	0.312	0.0003
Pipe material	0.9839	0.167	0.0015	0.101	0.0001
Pipe diameter	0.9508	0.161	0.0014	0.0971	0.0001
Pipe length	0.4493	0.0761	0.0007	0.0458	0.0000

**Table 11:** Five samples for resiliency of pipes with inputs and outcome.

	Object ID	162	634	2148	2705	31,938
Parameter						
Pipe age		42	61	68	12	19
Pipe material		Moderate	Moderate	Moderate	Moderate	Excellent
Pipe diameter (mm)		1650	250	300	375	900
Pipe length (m)		247.25	101.803	86.35	86.685	25.729
Land use type		Poor	Moderate	Moderate	Moderate	Moderate
Earthquake magnitude		Medium	Medium	Medium	Medium	Medium
Financial resource		High	High	High	High	High
Approachability		High	High	High	High	High
Degree of damage		Medium	Medium	Medium	Medium	Medium
Structural monitoring		Poor	Poor	Poor	Poor	Poor
Resiliency (%)		42.63	48.89	51.41	57.13	62.20

## Data Availability

The anonymized data are available from the corresponding author.
